# Fruit flies step out

**DOI:** 10.7554/eLife.00450

**Published:** 2013-01-08

**Authors:** Ronald L Calabrese

**Affiliations:** 1 **Ronald L Calabrese** is an *eLife* reviewing editor, and is in the Department of Neuroscience and Behavioral Biology, Emory University, Atlanta, United Statesrcalabre@biology.emory.edu

**Keywords:** walking behaviour, coordination, neurophysiology, sensory feedback, gait analysis, motor neuron, D. melanogaster

## Abstract

A method that can analyse the movements of *Drosophila* as they walk is a valuable addition to the tools available to neurobiologists, and has already led to insights into the interplay of central networks and sensory feedback in this model organism.

**Related research article** Mendes CS, Bartos I, Akay T, Márka S, Mann RS. 2013. Quantification of gait parameters in freely walking wild type and sensory deprived *Drosophila melanogaster*. *eLife* **2**:e00231. doi:10.7554/eLife.00231

**Image** A fruit fly as seen with FlyWalker software; the fly's footprints are illuminated

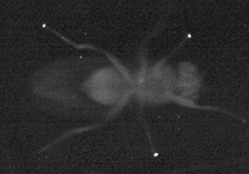

Movement—whether by water, air or land—is central to animal life, enabling organisms to find food and mates and to escape from predators. In the early twentieth century, the physiologist T Graham Brown identified complex networks of neurons in the spinal cords of cats that coordinated walking and other rhythmic movements (Brown, 1911, 1914). However, it was only when these networks—which are also called central pattern generators—were rediscovered in the early 1960s that their importance was fully appreciated (Wilson, 1961; Ikeda and Wiersma, 1964; Mulloney and Smarandache, 2010). From this point onwards, research into the generation of rhythmic activity by these networks and the influence of sensory feedback on this activity, has continued at a rapid pace (Marder et al., 2005; Marder and Bucher, 2007; Lamb and Calabrese, 2011).

In mammals, research into central pattern generators in the spinal cord has benefited from the advances in molecular biology afforded by the sequencing of the mouse genome (Goulding, 2009). Although our knowledge of *Drosophila* (fruit fly) genetics is arguably even more extensive, the small size of fruit flies has traditionally made physiological studies difficult. However, this situation is starting to change, and several groups have explored locomotion (Berni et al., 2012; Heckscher et al., 2012) and its development (Crisp et al., 2012) in fruit fly maggots.

Now, writing in *eLife*, César Mendes of Columbia University and colleagues, working in the laboratory of Richard Mann, report the development of new technology that can be used to track and to quantify walking in adult fruit flies (Mendes et al., 2013). Until now, this had required time-consuming manual frame-by-frame video analysis, which is a difficult process to automate (Wosnitza et al., 2012). Exploiting an optical phenomenon known as frustrated total internal reflection (fTIR), Mendes and co-workers have developed an imaging method and associated analysis software that can rapidly analyze the footprints (tarsal contacts) and body position of a fly as it walks (Figure 1). As well as revealing the insect's speed and the distance covered, the software extracts key parameters such as the degree to which limb movements are coordinated, thus allowing the insect's gait to be classified. Similar technology is already available for tracking the movements of larger animals such as rats (http://www.noldus.com/animal-behavior-research/products/catwalk), but the Columbia team is the first to scale it down to fly-like dimensions.

Insects generally employ either a tripod gait—in which three legs swing forward and the other three push backward against the ground (stance)—or a tetrapod gait, in which two legs are in swing and the remaining four are in stance. In addition to these, insects display other ‘non-canonical’ gaits that are harder to classify.

**Figure 1.**
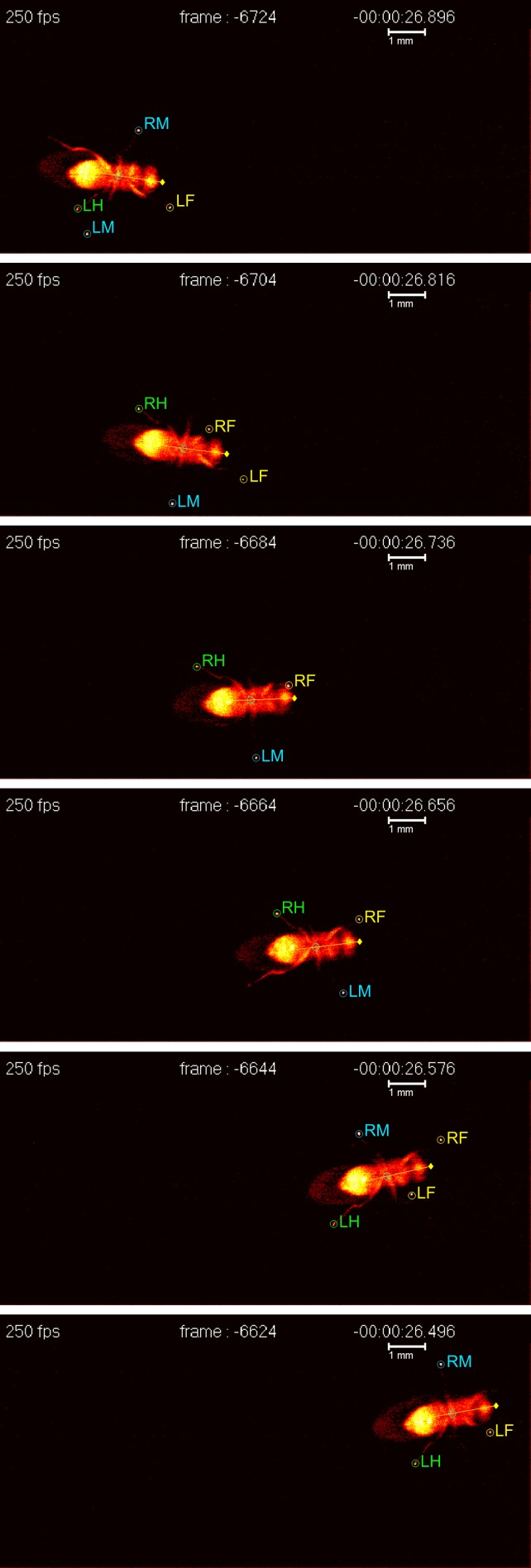
A fruit fly walking on a transparent surface. The fly was filmed from below at 250 frames per second using the FlyWalker system (biooptics.markalab.org/FlyWalker/); the frames shown here were recorded 0.08 seconds apart. A tripod stance can be seen in the third, fourth and sixth images of this series. The six legs of the fly are labelled RH (right hind), RM (right middle), RF (right front), LH (left hind), LM (left middle) and LF (left front).

FIGURE CREDIT: CS MENDES and I BARTOS.

Mendes et al. find that flies, unlike vertebrates, do not show abrupt transitions from one gait to another as they increase or decrease their speed: rather, they employ a mixture of gaits that changes with their walking speed: faster flies tend to spend more time in a tripod configuration, whereas slower flies spend more time in tetrapod or non-canonical configurations. These differences led Mendes et al. to suggest that flies use distinct neural programs for walking at different speeds.

However, the most interesting results in the current work concern the role of sensory feedback in modulating the output of central pattern generators. To examine this interaction, the Columbia team used genetically modified flies that either lack all leg proprioceptors—sensors that detect the length and tension of muscles and the angle of joints, which together provide information about a limb's position in space—or a specific class of stretch receptors known as chordotonal organs. Both types of mutant fly walked more slowly than wild-type flies, and showed a more uneven gait with footprints that were less precisely aligned. Nevertheless, the mutant flies maintained a typical tripod gait and near normal coordination of limb movements with respect to body segment and side. It seems therefore that proprioception is not essential for coordinated walking, but walking is less precise without it.

Being deprived of sensory feedback was more disruptive for mutant flies that walked slowly than it was for faster individuals. Indeed, in some respects, the behaviour of feedback-deprived flies resembled that of wild-type flies walking at high speed. These observations suggest that flies walking at slow–medium speeds use distinct neural programs from those walking at faster speeds, and that flies walking at fast speeds are less dependent on sensory feedback.

The results of this study complement work on other insects. In the slow-moving stick insect (*Carausius*), sensory feedback from legs in the stance phase appears to be required for inter-leg coordination (Büschges 2012), whereas this does not seem to be the case in the fruit-fly experiments at Columbia. To account for this apparent discrepancy, Mendes et al. speculate that when all six legs are equally impaired, flies resort to using the central pattern generator alone, which is sufficient to execute the tripod gait. Other recent studies on adult flies, in which single legs were amputated, are consistent with this speculation (Wosnitza et al., 2012).

The future therefore seems bright for dissection of the neuronal networks, and their sensory partners, that generate rhythmic movements in *Drosophila*. Although work on more traditional invertebrate preparations—in which neurons are readily accessible for electrophysiology (Marder et al., 2005; Marder and Bucher, 2007; Lamb and Calabrese, 2011)—will continue to guide the way for now, I expect that as their genetic and molecular armamentarium expands, flies will come to play a seminal role in neuronal network analysis.
